# A case report of entrapment of PentaRay catheter in the mechanical mitral valve: a known complication experienced anew

**DOI:** 10.1093/ehjcr/ytae194

**Published:** 2024-04-16

**Authors:** Hui-Qiang Wei, Fangzhou Liu, Yumei Xue, Shulin Wu, Xianzhang Zhan

**Affiliations:** Department of Cardiology, Guangdong Cardiovascular Institute, Guangdong Provincial People's Hospital, Guangdong Academy of Medical Sciences, No. 106 Zhongshan 2nd Road, Yuexiu District, 510000 Guangzhou, China; Department of Cardiology, Guangdong Cardiovascular Institute, Guangdong Provincial People's Hospital, Guangdong Academy of Medical Sciences, No. 106 Zhongshan 2nd Road, Yuexiu District, 510000 Guangzhou, China; Department of Cardiology, Guangdong Cardiovascular Institute, Guangdong Provincial People's Hospital, Guangdong Academy of Medical Sciences, No. 106 Zhongshan 2nd Road, Yuexiu District, 510000 Guangzhou, China; Department of Cardiology, Guangdong Cardiovascular Institute, Guangdong Provincial People's Hospital, Guangdong Academy of Medical Sciences, No. 106 Zhongshan 2nd Road, Yuexiu District, 510000 Guangzhou, China; Department of Cardiology, Guangdong Cardiovascular Institute, Guangdong Provincial People's Hospital, Guangdong Academy of Medical Sciences, No. 106 Zhongshan 2nd Road, Yuexiu District, 510000 Guangzhou, China

**Keywords:** Atrial tachycardia, Mechanical valve, High-density mapping, PentaRay catheter, Case report

## Abstract

**Background:**

Complex atrial tachycardia (AT) is commonly observed in patients with cardiac surgery. High-density mapping is widely adopted for catheter ablation of complex AT in patients with cardiac surgery. Several case reports have described that PentaRay mapping catheter can be trapped in the mechanical valve and sheared off and successful retrieval of the spline by a snare system. We described a rare case in which PentaRay mapping catheter spline was successfully retrieved from the distal anterior tibial artery by direct syringe suction via the diagnostic catheter following entrapment in the mechanical mitral valve (MV) and rupture of the spline.

**Case summary:**

A 70-year-old female with mechanical bileaflet MV underwent catheter ablation for AT. During mapping in left atrium, the catheter was entrapped in mechanical MV and sheared off. We attempted to release the entrapped the spline by advancing the ablation catheter towards the stuck disc and pushing on the hinge portion of the disc with the catheter tip. The stuck and closed disc was opened, and the deeply entrapped spline was released. However, the entrapped PentaRay spline floated through the Valsalva sinus and strayed into the distal left anterior tibial artery. Fortunately, we successfully retrieved the spline from the distal anterior tibial artery by direct syringe suction instead of a snare system.

**Discussion:**

The possibility of the entrapment and subsequent rupture of the spline should always be considered during mapping the site close to mechanical valve. A rapid retrieval of embolized material should be carried out. If the spline strays into the distal and small artery in which the snare system is difficult to advance, a direct syringe suction via the diagnostic catheter may be attempted.

**Learning points**

- PentaRay catheter provides rapid and accurate high-density mapping that facilitates the mapping of complex atrial arrhythmias, but may be at risk for entrapment in mechanical valve.
- Sheared splines can stray into critical blood vessels, and a rapid retrieval of embolized material should be carried out.
- If the spline strays into the distal and small artery in which the snare system is difficult to advance, a direct syringe suction via the diagnostic catheter may be attempted.

## Introduction

Atrial tachycardia (AT) is commonly observed in patients with cardiac surgery. The mechanism of AT is most often macro-re-entry originating from scars at the surgical access site, at the insertion sites for the bypass cannulation, or around the implanted valve itself.^1^ High-density mapping is widely adopted for catheter ablation of AT in patients with cardiac surgery. However, several case reports have described that PentaRay mapping catheter can be trapped in the mechanical valve owing to structural problems.^2–4^ To date, there have been no reports of a PentaRay mapping catheter being entrapped, being sheared, and then straying into the distal anterior tibial artery. Here, we described a complex AT case after mitral valve (MV) replacement in which PentaRay mapping catheter spline was successfully retrieved from the distal anterior tibial artery by direct syringe suction via the diagnostic catheter without a snare system following entrapment in the mechanical MV and rupture of the spline.

## Summary figure

**Figure:**
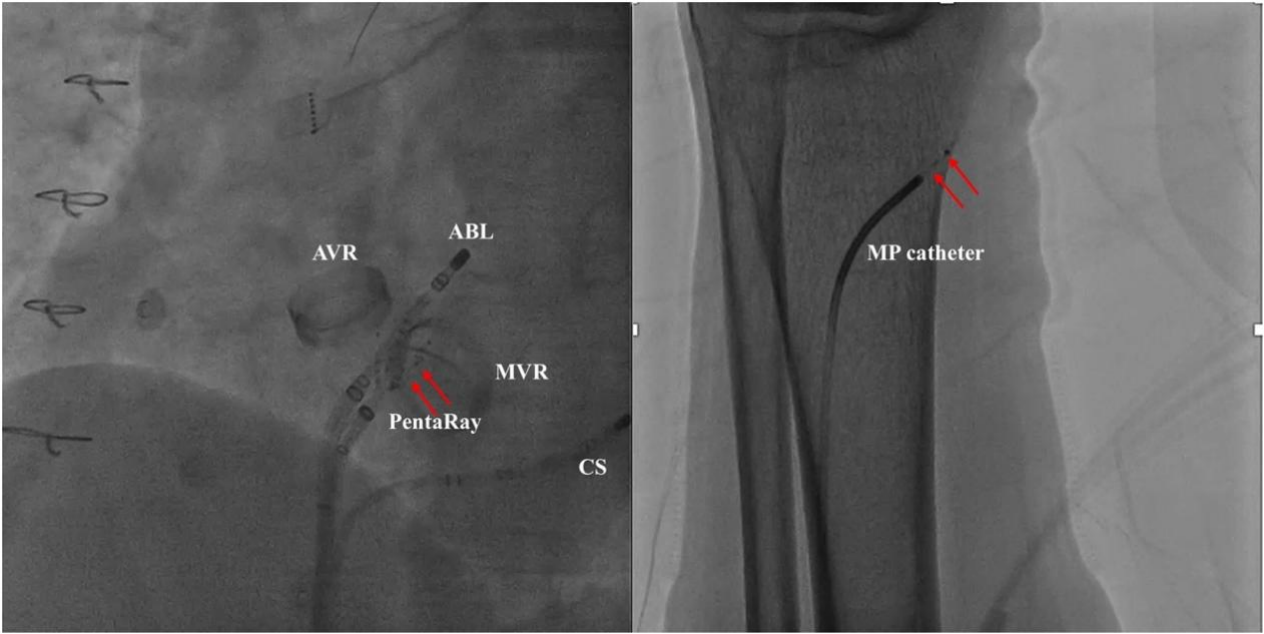
Left: fluoroscopic image in LAO demonstrating the entrapment of one of the spline of PentaRay catheter in the mechanical MV. Right: localization of the missing element (red arrow) in the left anterior tibial artery and successful retrieval by direct syringe suction via the MP diagnostic catheter.

## Case presentation

A 70-year-old female with history of symptomatic palpitations was referred to our centre for catheter ablation of AT. The patient had undergone MV and aortic valve (AV) replacement (St. Jude Medical, St. Paul, MN) more than 20 years earlier due to rheumatic heart disease. Antiarrhythmic drugs such as amiodarone failed to control the heart rate and the patient was still symptomatic. Cardiac auscultation demonstrated metallic ticking sound at mitral and aortic area. Her lungs were clear and had no signs of heart failure. Pre-operative transthoracic echocardiography showed that the bileaflet prosthetic function remained normal and left ventricular function was preserved, with an estimated systolic left ventricular function of 68%.

**Figure 1:**
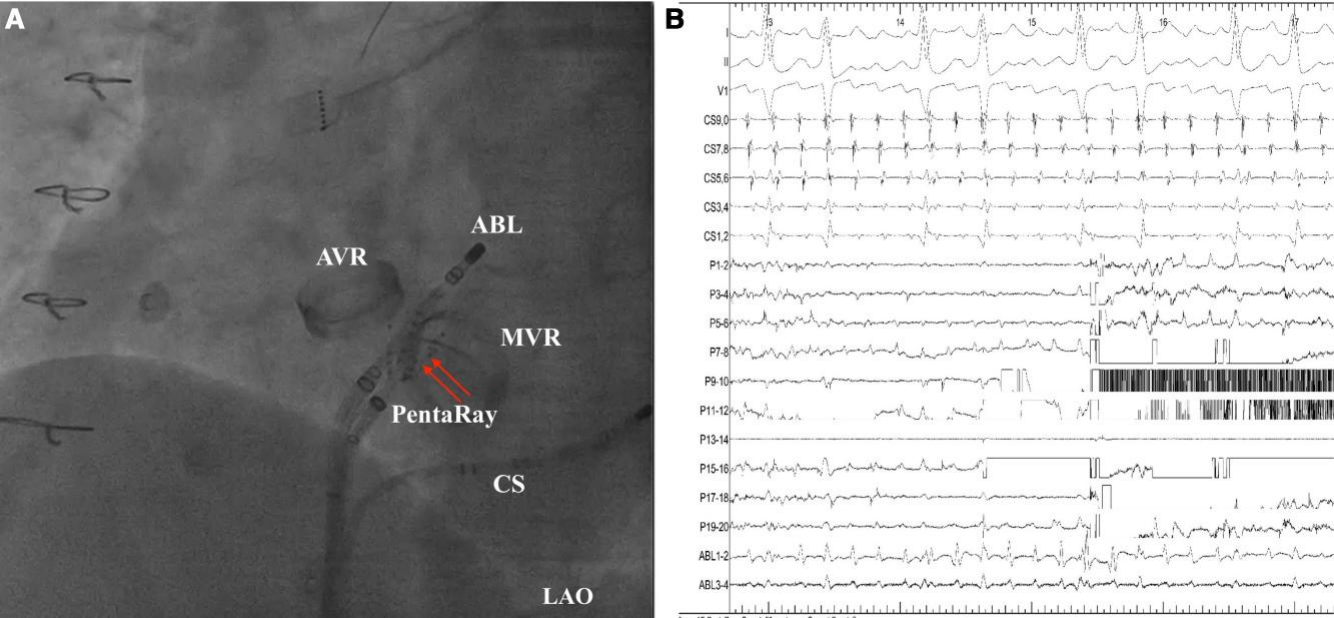
(*A*) Fluoroscopic image in LAO demonstrating the entrapment of one of the spline of PentaRay catheter in the mechanical MV. (*B*) Intracardiac electrocardiogram showed the signal interference in PentaRay catheter. ABL, ablation catheter; AVR, aortic valve replacement; CS, coronary sinus; LAO, left anterior oblique; MV, mitral valve; MVR, mitral valve replacement.

**Figure 2:**
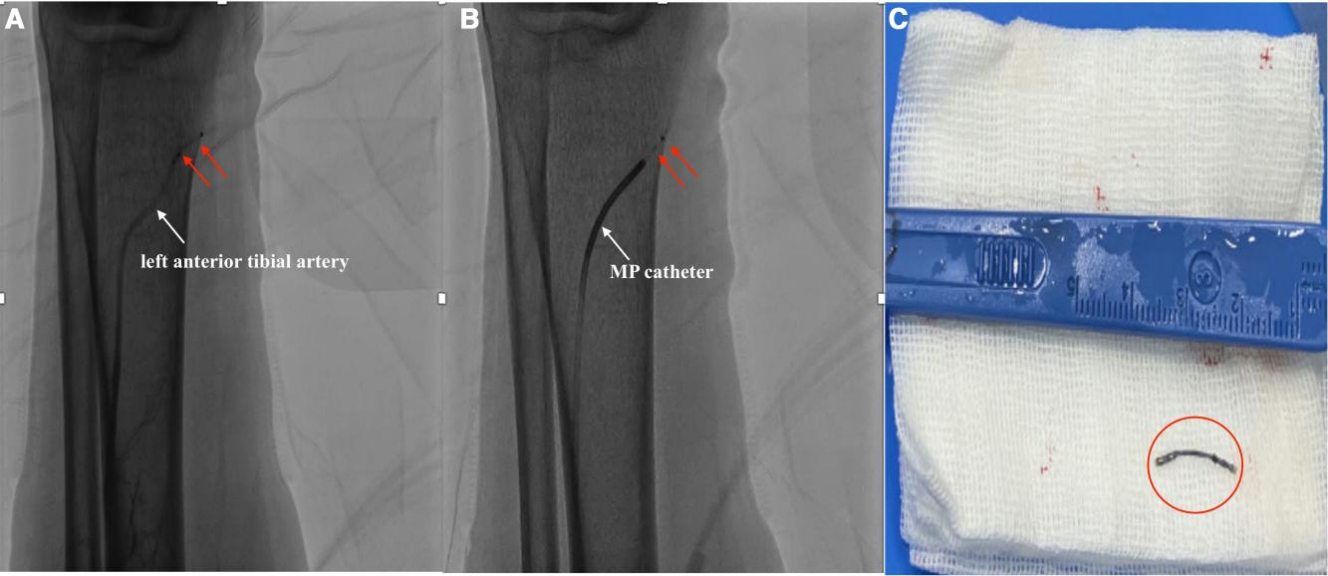
(*A*) Localization of the missing element in the left anterior tibial artery. (*B*) Successful retrieval by direct syringe suction via the MP diagnostic catheter. (*C*) Retrieval of the spline. MP, multipurpose.

After intracardiac thrombi were excluded by transoesophageal echocardiography, the procedure was performed under local anaesthesia. Three-dimensional electroanatomical mapping using multipolar catheter (PentaRay, Biosense Webster, Diamond Bar, CA) and radiofrequency ablation application using an ablation catheter (ThermoCool SmartTouch; Biosense Webster) were performed. Venous femoral puncture was performed and a decapolar catheter was advanced in the coronary sinus as a reference. Activation mapping in right atrium (RA) using PentaRay catheter demonstrated that RA was not involved in the tachycardia. Double transseptal access was achieved and two non-steerable sheaths were positioned in the left atrium (LA). Despite cautious manipulation of the PentaRay in the anterior wall of LA, the catheter was entrapped in the septal aspect of the mechanical MV, a fluoroscopic image demonstrated the fixed ipsilateral disc of the mechanical MV, suggesting that one of splines of the PentaRay was entrapped in the hinge portion between the disc and the orifice ring (*Figure 1*). The catheter was easily retracted into the sheath but one of the splines had sheared off. Therefore, the ablation procedure was abandoned. We attempted to release the entrapped the spline by advancing the ablation catheter towards the stuck disc and pushing on the hinge portion of the disc with the catheter tip. The stuck and closed disc was luckily opened, and the deeply entrapped spline was released. Immediately thereafter, the entrapped spline floated through the Valsalva sinus. After an initial check to rule out cerebral embolization, the missing element was located in the distal left anterior tibial artery. We attempted to extract the spline by direct syringe suction via a multipurpose (MP) diagnostic catheter instead of grasping the spline with a snare because it might be difficult to advance the snare system to the distal anterior tibial artery. The MP diagnostic catheter was advanced to the distal left anterior tibial artery on the guidewire smoothly via a 6 F arterial femoral sheath. Next, the spline was successfully retrieved into the MP catheter following direct syringe suction (*Figure 2*) (see Supplementary material online, *Video*). No complications occurred during the procedure. Post-operative echocardiography was performed and demonstrated normal function of the mechanical valve. The patient had no symptoms of right lower extremity and continued to intake antiarrhythmic drugs at the follow-up period of three months.

## Discussion

This case illustrates a serious complication that may occur when using the PentaRay mapping catheters in patients with mechanical valves. Entrapment of PentaRay catheter in mechanical valves is a known complication and has been reported for many years.^2,4^ However, patients with prosthetic valve replacement have a high incidence of complex atrial arrhythmias, and PentaRay is widely used in clinical practice to gain much information and eliminate arrhythmias effectively. This approach seems attractive in this type of patients with atrial arrhythmias. However, extreme caution should be taken when mapping close to mechanical valves. In the current literature, there are several reports of successfully opening a stuck valve and releasing an entrapped PentaRay spline by pushing the fixed disc with an ablation catheter.^2,3^ We attempted similar approach and successfully opened the stuck valve. However, the entrapped PentaRay spline floated through the Valsalva sinus and strayed into the distal left anterior tibial artery. Luckily, we successfully retrieved the spline from the distal anterior tibial artery by direct syringe suction instead of a snare system. The patient suffered no significant sequelae. This is the first report of a torn PentaRay spline straying into the distal anterior tibial artery and successful retrieval by direct syringe suction. To avoid this complication, mapping in the area adjacent to the mechanical valve can be carried out with the ablation catheter instead of the multipolar mapping catheter. Furthermore, immediate identification of the location and recovery of the torn spline are essential. If the spline strayed into the distal and small artery in which the snare system was difficult to advance, a direct syringe suction via the diagnostic catheter might be attempted.

## Conclusion

PentaRay catheter provides rapid and accurate high-density mapping that facilitates the mapping of complex atrial arrhythmias. However, the possibility of the entrapment and subsequent rupture of the spline should always be considered during mapping the site close to mechanical valve. A rapid retrieval of embolized material should be carried out. If the spline strays into the distal and small artery in which the snare system is difficult to advance, a direct syringe suction via the diagnostic catheter may be attempted.

## Supplementary Material

ytae194_Supplementary_Data

## Data Availability

The data that support the findings of this study are available from the authors upon reasonable request.

## Lead author biography

Dr Wei is a cardiac electrophysiology specialist at the hospital of Guangdong Cardiovascular Institute. His specialties include cardiovascular disease and internal medicine.

## Supplementary material

**Consent:** The authors confirm that written consent for submission and publication of this case report including images and associated text has been obtained from the patient in line with COPE guidance.

**Funding:** None declared.
